# Impact of Neuromuscular Electrical Stimulation on Biological Markers in Critically Ill Patients: A Systematic Review and Meta‐Analysis

**DOI:** 10.1155/ccrp/5382735

**Published:** 2026-01-20

**Authors:** Amanda de Oliveira Santos, Matheus Cardoso Santos, Maíra Avila Fontes Trindade, Danielle Alves de Andrade Rebouças, Carlos José Oliveira de Matos, Fernanda Oliveira de Carvalho, Paulo Ricardo Martins-Filho, Érika Ramos Silva

**Affiliations:** ^1^ Postgraduate Program in Applied Health Sciences, Federal University of Sergipe, Av. Gov. Marcelo Déda, Lagarto, 49400-000, Sergipe, Brazil, ufs.br; ^2^ Centre for Robotics & Intelligent Systems (CRIS), University of Limerick, Limerick, V94 T9PX, Munster, Ireland, ul.ie; ^3^ Investigative Pathology Laboratory, Federal University of Sergipe, Av. Gov. Marcelo Déda, Lagarto, 49400-000, Sergipe, Brazil, ufs.br

**Keywords:** biomarkers, critical care, critical illness, electric stimulation, hospitalization, intensive care unit, meta-analysis, systematic review

## Abstract

**Purpose:**

Neuromuscular electrical stimulation (NMES) has been increasingly used to preserve or restore neuromuscular function in critically ill patients. However, its effects on inflammatory biomarkers and its safety require to be fully elucidated. This study aimed to analyze the available evidence on the impact of NMES on biological markers in critically ill patients.

**Methods:**

This systematic review followed a preregistered protocol (PROSPERO: CRD42023424413). A comprehensive search was conducted in PubMed, EMBASE, Web of Science, Scopus, PEDro, CENTRAL, and Google Scholar to identify randomized controlled trials (RCTs) comparing NMES with control interventions and reporting outcomes related to biological markers.

**Results:**

Ten RCTs were included in this review. Meta‐analyses revealed a significant acute increase in interleukin‐10 levels (SMD: 0.60; 95% CI: 0.11 to 1.08; *p* = 0.02) and a delayed reduction in serum C‐reactive protein levels (SMD: −0.74; 95% CI: −1.09 to −0.40; *p* < 0.0001) following NMES application.

**Conclusions:**

Available evidence suggests that NMES can modulate systemic inflammation in mechanically ventilated critically ill patients, with early anti‐inflammatory effects (IL‐10 elevation) and subsequent attenuation of inflammation (CRP reduction). These findings support the safety of NMES during active phases of critical illness. Further high‐quality RCTs are warranted to standardize stimulation protocols, characterize biomarker dynamics, and elucidate the underlying mechanisms to guide evidence‐based clinical use.

## 1. Introduction

Patients admitted to intensive care units (ICU) require comprehensive care, experience restricted mobility, and frequently necessitate advanced life support interventions [1]. The combination of systemic inflammation, neuroendocrine dysregulation, and prolonged immobilization contributes to muscle wasting, driven by a negative protein balance [2]. Additional risk factors, such as prolonged mechanical ventilation, administration of sedatives and corticosteroids, hyperglycemia, parenteral nutrition, and extended bed rest, further increase the likelihood of developing the condition known as ICU‐acquired weakness (ICU‐AW) [3]. ICU‐AW is a neuromuscular dysfunction [4] that affects approximately 48% of critically ill patients [2] and is characterized by muscle atrophy, decreased reflexes, and limb paresis or paralysis [5]. Although the underlying mechanisms of muscle deterioration are not fully elucidated, evidence suggests that the interaction between physical inactivity, proinflammatory cytokines, and oxidative stress plays a central role in the development of muscle dysfunction [6].

Neuromuscular electrical stimulation (NMES) has emerged as a promising therapeutic intervention to mitigate muscle degradation in critically ill patients, particularly when active mobilization is not feasible [7]. NMES delivers low‐frequency electrical currents through surface electrodes to elicit involuntary muscle contractions, independent of voluntary motor control [8]. This allows muscle activation even in deeply sedated or immobilized patients. Studies employing rigorous methodologies, including randomized controlled trials (RCTs), have demonstrated that NMES can preserve muscle strength and mass [9], reduce the duration of mechanical ventilation, shorten ICU stay [8], and potentially lead to modest improvements in functional outcomes [10]. Moreover, NMES‐induced contractions stimulate the release of myokines and anti‐inflammatory cytokines, indicating beneficial effects at the molecular level. Wahl et al. [11], for instance, reported that NMES activates muscle tissue to secrete cytokines, thereby enhancing muscle protein synthesis and preserving structural integrity. These findings support the integration of NMES into early rehabilitation protocols in the ICU setting.

Technical factors, including protocol specifications, impedance variability due to edema or adiposity, and the electrode‐to‐muscle ratio, as well as potential adverse effects, may influence the contractile response to NMES, impacting its effectiveness [12]. The safety of this intervention can be monitored through metabolic, hemodynamic, ventilatory, inflammatory, tissue, and muscle evaluations [13]. Although some studies have investigated the impact of NMES on biological markers in ICU patients, further clarification is needed regarding the effects of this therapeutic modality. To date, no systematic review with meta‐analysis has been conducted to elucidate the effects of NMES on inflammatory markers in critically ill patients. It is anticipated that synthesizing the available evidence on this topic will enhance the understanding of NMES‐induced biological responses and inform clinical decision‐making regarding its use in critically ill populations.

## 2. Methods

This systematic review with meta‐analysis of RCTs followed the Preferred Reporting Items for Systematic Review and Meta‐Analysis (PRISMA) statement [14]. The systematic review protocol was registered in the International Prospective Register of Systematic Reviews (PROSPERO) on May 19, 2023, under registration number CRD42023424413.

### 2.1. Search Strategy

The search strategy was conducted across the following databases: PubMed, EMBASE, PEDro, Web of Science, Scopus, and Cochrane Central Register of Controlled Trials (CENTRAL). Additionally, gray literature was examined through manual searches of reference lists of included studies, as well as databases such as Digital Library of Thesis and Dissertations (BDTD), ProQuest, and Google Scholar (first 100 results). No restrictions were applied regarding year of publication or language. The search strategies were adapted for each database (supporting file (available here)) and performed on February 10, 2025.

### 2.2. Study Selection and Eligibility Criteria

Two independent investigators (Amanda de Oliveira Santos and Danielle Alves de Andrade Rebouças) screened the retrieved studies by titles and abstracts. Full texts of potentially eligible articles were assessed according to the predefined eligibility criteria. Disagreements were resolved through consensus or by consultation with a third reviewer (Érika Ramos Silva).

Studies were included if they met the following criteria: (a) Population: adult patients (≥ 18 years), of any gender, race, or ethnic origin, admitted to an ICU for any medical condition; (b) Intervention: NMES applied either as a standalone therapy or in combination with standard care; (c) Comparison: absence of NMES, placebo, sham stimulation, routine intervention, mobilization protocols, or other motor physiotherapy modalities; (d) Outcomes: biological markers; (e) Study type: RCTs.

Exclusion criteria were as follows: (a) Population: studies involving animal models or patients not admitted to an ICU; (b) Intervention: therapies aimed solely at analgesic effects; (c) Comparison: studies combining NMES with another therapeutic modality, using the contralateral hemibody as control, or applying NMES with different current parameters between groups; (d) Outcomes: studies that did not assess biological markers and focused exclusively on functional or muscle strength outcomes; (e) Study type: observational studies, systematic reviews, editorials, book chapters, conference abstracts, pilot studies, or case reports.

### 2.3. Data Extraction

Extracted data included study characteristics (e.g., author, country, and year of publication), study objectives, intervention and control group descriptions, sample size, participant characteristics (e.g., number, age, gender), intervention details (e.g., frequency, pulse width [PW], rise time, sustainment, descent, on/off time, intensity, number of sessions, target muscles, equipment used), assessed biomarkers, and study findings. Means and standard deviations (SD) of biomarker levels were extracted from each study. When numerical data were unavailable in tables or text, they were obtained using the WebPlotDigitizer tool [15]. Study authors were contacted when necessary to clarify or obtain additional data.

### 2.4. Assessment of Risk of Bias in Included Studies

Risk of bias was independently assessed by two reviewers (Danielle Alves de Andrade Rebouças and Amanda de Oliveira Santos) following the Cochrane Risk of Bias Tool for RCTs [16]. The following seven domains were evaluated: sequence generation and allocation concealment (selection bias), blinding of participants and researchers (performance bias), outcome assessment (detection bias), incomplete outcome data (attrition bias), selective outcome reporting (reporting bias), and other potential sources of bias. Each domain was classified as having low, unclear, or high risk of bias. The assessment and visual representation were performed using Review Manager (RevMan) version 5.4, developed by The Cochrane Collaboration.

### 2.5. Data Analysis

Each biomarker was analyzed separately and categorized as either proinflammatory or anti‐inflammatory. A meta‐analysis was performed when at least two studies assessed the same biomarker. All dependent variables were continuous, and the standardized mean difference (SMD) was calculated by dividing the difference in means between groups by the pooled SD.

The choice of meta‐analytic model was guided by the degree of heterogeneity, assessed using the *I* ^2^ statistic [17]. In the presence of heterogeneity, the DerSimonian and Laird random‐effects model was applied; otherwise, a fixed‐effect model was used. In fixed‐effects analyses, weights were assigned as the inverse of the variance (the square of the standard error, SE), while in random‐effects analyses, both within‐ and between‐study variances were considered.

Effect sizes were reported with 95% confidence intervals (CIs) and represented in forest plots. For proinflammatory biomarkers, a negative SMD indicated a reduction in biomarker levels associated with NMES. For anti‐inflammatory biomarkers, a positive SMD indicated an increase in biomarker levels. A two‐tailed *p*‐value < 0.05 was considered statistically significant. All statistical analyses were conducted using Review Manager (RevMan), version 5.4.

### 2.6. Certainty of Evidence

The quality of evidence and strength of recommendations were assessed using the GRADE (Grading of Recommendations, Assessment, Development and Evaluations) framework [18]. This assessment considered limitations in study design, inconsistency, indirectness, imprecision, and magnitude of effect. Due to the limited number of studies per biomarker, publication bias was not formally assessed. The overall certainty of evidence was rated as high, moderate, low, or very low.

## 3. Results

**Figure 1:**
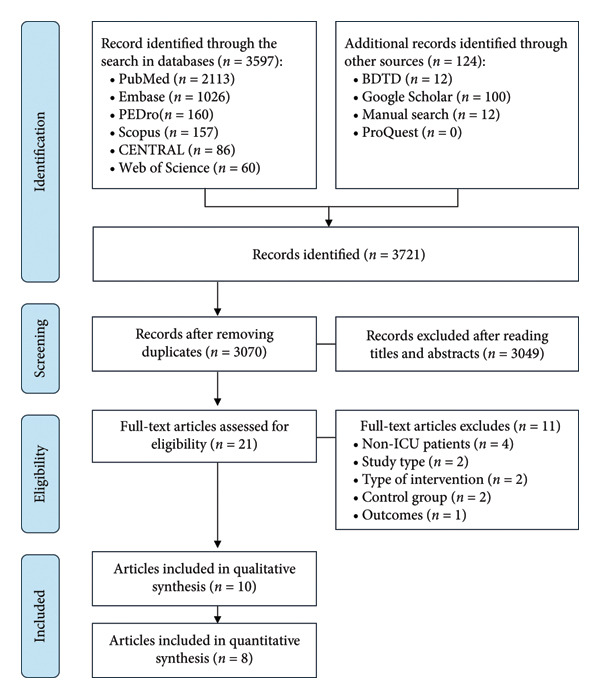
PRISMA flow diagram for study selection.

A total of 3721 articles were initially identified, of which 651 were duplicates and subsequently removed. Among the remaining 3070 studies, 3049 were excluded based on title and abstract screening, and 21 were considered potentially eligible for full‐text analysis. Following detailed evaluation, 11 studies were excluded for not meeting the inclusion criteria. Ultimately, 10 articles were included in the qualitative synthesis, and eight of these were eligible for quantitative analysis. A flowchart illustrating the study selection process is presented in Figure 1.

### 3.1. Study Characteristics

Ten studies met the inclusion criteria. Detailed information regarding study populations, objectives, sample sizes and characteristics, interventions, biomarkers assessed, and key findings is provided in Table 1. The geographical distribution of the studies was as follows: three conducted in Brazil [19–21], one each in Turkey [22], China [23], Japan [24], Australia [25], Italy [26], Greece [27], and France [28].

**Table 1:** Summary of included randomized controlled trials evaluating NMES effects on biological markers in critically ill patients.

Author (Year), country	Objectives	Groups (*n*)	Sample size and patient characteristics	Markers assessed	Results and conclusion
Abdellaoui et al. (2011), France [28]	To investigate the effects of a 6‐week NMES program on muscle strength and muscular oxidative stress during recovery from acute exacerbation of COPD	Intervention: G1 = NMES + rehabilitation program (*n* = 9) Control: G2 = Sham (*n* = 6)	*n* = 15 Patients with COPD admitted to the ICU due to acute exacerbation Sex (M/F) = G1: 7/2; G2: 6/0 Age (median; IQR): G1: 59 (57–69) G2: 67 (59–72)	Total protein and MyHC carbonylation 4‐HNE‐modified proteins TBARs Muscle fiber type	G1: reduction in total protein levels and MyHC carbonylation (indicative of reduced oxidative stress); increased proportion of type I muscle fibers
Kayambu et al. (2015), Australia [25]	To determine whether early physical rehabilitation improves physical function and associated outcomes in patients with sepsis	Intervention: G1 = NMES + mobilization (*n* = 26) Control: G2 = Conventional physiotherapy (*n* = 24)	*n* = 50 Patients with sepsis admitted to the ICU Sex (M/F) = G1: 8/18; G2: 10/14 Age (median; IQR): G1: 62.5 (30–83) G2: 65.5 (37–85)	IL‐6 IL‐10 TNF‐α Lactate	G1: increase in IL‐10 levels
Akar et al. (2017), Turkey [22]	To evaluate the impact of active mobilization and NMES on weaning, hospital discharge, and inflammatory mediators in COPD patients receiving mechanical ventilation	Intervention: G1 = NMES + active mobilization (*n* = 10) G2 = NMES only (*n* = 10) Control: G3 = Active mobilization only (*n* = 10)	*n* = 30 Patients with COPD and acute respiratory failure under IMV Sex (M/F) = G1: 4/6; G2: 6/4; G3: 5/5 Age (mean ± SD): G1: 70 ± 12.3 G2: 62.8 ± 6.8 G3: 68 ± 17.8	CRP IL‐6 IL‐8 IL‐10 TNF‐α	G1: reduction in IL‐6 and IL‐8 levels G2: reduction in CRP and IL‐8 levels
Koutsioumpa et al. (2018), Greece [27]	To assess whether the application of transcutaneous electrical neuromuscular stimulation reduces the incidence or severity of critical illness‐related myopathy in ICU patients	Intervention: G1 = TENMS + conventional physiotherapy (*n* = 38) Control: G2 = conventional physiotherapy (*n* = 42)	*n* = 80 Critically ill patients with myopathy Sex (M/F) = G1: 26/12; G2: 34/8 Age (mean ± SD): G1: 64 ± 12.4 G2: 66 ± 13.1	CRP Glucose Creatine kinase	No significant differences were observed between groups
Silva et al. (2019), Brazil [20]	To assess the effects of an NMES protocol on muscle architecture, neuromuscular electrophysiological disturbance, muscle strength, plasma systemic inflammation, catabolic responses, and clinical outcomes	Intervention: G1 = NMES + conventional physiotherapy (*n* = 30) Control: G2 = conventional physiotherapy (*n* = 30)	*n* = 60 Patients undergoing mechanical ventilation following traumatic brain injury Sex (M/F) = G1: 26/4; G2: 26/4 Age (mean; 95%CI): G1: 33 (29–37) G2: 30 (27–33)	TGF‐β IGF‐1 IL‐1β IL‐6 IL‐8 IL‐10 TNF‐α	No significant differences were observed between groups
França et al. (2020), Brazil [19]	To evaluate the effects of NMES and PCE on oxidative stress and inflammatory mediators in critically ill patients under mechanical ventilation	Intervention: G1 = NMES (*n* = 9) G2 = NMES + PCE (*n* = 7) G3 = PCE (*n* = 9) Control: G4 = no intervention (*n* = 10)	*n* = 35 Critical ill patients under mechanical ventilation Sex (M/F) = 37.2%/62.8% Age (mean ± SD): G1: 64.1 ± 18.2 G2: 60.1 ± 27.2 G3: 64.2 ± 10.5 G4: 56.8 ± 12.8	NO (C+) NO (C−) IFN‐γ IL‐6 IL‐10 TNF‐α	G1: reduction in nitrate oxidative levels G3: reduction in nitrate oxidative and TNF‐α levels
Nakanish et al. (2020), Japan [24]	To investigate whether NMES prevents muscle atrophy in the upper and lower limbs and improves physical function	Intervention: G1 = NMES + mobilization protocol (*n* = 17) Control: G2 = mobilization protocol (*n* = 19)	*n* = 36 Adult patients on mechanical ventilation for > 48 h and hospitalized in the ICU for > 5 days Sex (M/F) = G1: 12/5; G2: 12/7 Age (mean ± SD): G1: 73 ± 3 G2: 66 ± 3	Amino acid levels Branched‐chain amino acids (BCAA)	G1: Reduction in BCAA levels
Bao et al. (2022), China [23]	To compare the efficacy and safety of NMES in preventing muscle atrophy in ICU patients without nerve injury	Intervention: G1 = NMES in gastrocnemius and tibialis anterior + active and passive mobilization (*n* = 20) G2 = NMES in gastrocnemius + active and passive mobilization (*n* = 20) Control: G3 = Active and passive mobilization (*n* = 20)	*n* = 60 ICU patients without nerve injury Sex (M/F) = G1: 14/6; G2: 18/2; G3: 19/1 Age (mean ± SD): G1: 52.8 ± 10.8 G2: 51.1 ± 17.6 G3: 52.5 ± 12.5	CRP PT Lactic acid	No significant differences were observed between groups
Lo Re et al. (2023), Italy [26]	To evaluate the effects of NMES combined with standard rehabilitation on preserving skeletal muscle mass and the secretion of myokines associated with cognitive outcomes in patients undergoing RM surgery	Intervention: G1 = NMES + routine rehabilitation (*n* = 23) Control: G2 = routine rehabilitation (*n* = 32)	*n* = 55 Patients undergoing RM surgery Sex (M/F) = G1: 21/2; G2: 25/7 Age (mean ± SD): G1: 63.1 ± 8.7 G2: 62.1 ± 12.2	Klotho FGF23 IL‐6 BDNF	G1: increase in IL‐6 and Klotho levels
Vieira et al. (2023), Brazil [21]	To evaluate the effects of an NMES protocol on muscle architecture, as well as on signaling mediators of muscle growth and systemic inflammation in patients with TBI	Intervention: G1 = NMES + early rehabilitation (*n* = 12) Control: G2 = early rehabilitation (*n* = 13)	*n* = 40 Patients undergoing MV Sex (M/F) = G1: 16/4; G2: 16/4 Age (mean ± SD): G1: 34.7 ± 11.2 G2: 36.5 ± 13.5	IGF‐1 IFN‐γ IL‐2 IL‐4 IL‐6 IL‐10 TNF‐α MMP‐2 MMP‐9	No significant differences were observed between groups

*Note:* TENMS, transcutaneous electrical neuromuscular stimulation; IL, interleukin; IFN‐γ, interferon gamma; IGF‐1, insulin‐like growth factor 1; NO (C+), nitric oxide in control‐stimulated cells; NO (C−), nitric oxide in unstimulated cells; MyHC, myosin heavy chain; 4‐HNE, 4‐hydroxynonenal; TBAR, thiobarbituric acid reactive substances; MMP, matrix metalloproteinase; NMES, neuromuscular electrical stimulation; M, male; F, female.

Abbreviations: ARF, acute respiratory failure; BDNF, brain‐derived neurotrophic factor; CK, creatine kinase; COPD, chronic obstructive pulmonary disease; CRP, C‐reactive protein; FGF23, fibroblast growth factor 23; ICU, intensive care unit; IMV, invasive mechanical ventilation; PCE, passive cycle ergometry; PT, prothrombin time; TGF‐β, transforming growth factor beta; TNF‐α, tumor necrosis factor alpha.

The studies included a total of 403 ICU patients, of whom 231 (50%) received NMES. Sample sizes ranged from 15 to 60 participants, and mean ages spanned from 30 to 73 years. All studies included both male and female participants, with a predominance of males (70.8%). All patients were under mechanical ventilation. Regarding clinical diagnoses, two studies evaluated critically ill patients with traumatic brain injury (TBI) [20, 21]; one on sepsis [25]; two on COPD [22, 28]; one on post‐coronary artery bypass graft surgery [26]; one on myopathy [27]; and three included critically ill patients without disease‐specific inclusion criteria [19, 23, 24].

### 3.2. Intervention

Two studies evaluated NMES as a standalone intervention compared to a control group [19, 22], while the remaining eight assessed NMES in combination with conventional exercise training versus control. The control groups received standard mobilization protocols [22–25], routine physiotherapy [20, 21, 26, 27], sham stimulation [28], or no intervention [19]. Various lower lib muscles were targeted, including quadriceps femoris [19, 21, 22, 24–28], gastrocnemius [20, 23, 26], anterior tibialis [20, 23, 25, 26], and hamstrings [20, 25]. Only three studies applied NMES to upper limb muscles: deltoid [22], brachioradialis [25], and biceps brachii [24].

Session durations ranged from 20 to 60 min, with frequencies of one to two sessions per day. One study [19] evaluated the acute effect after a single session. NMES frequencies ranged from 20 to 100 Hz, with 50 Hz being the most used (seven studies). PW ranged from 300 to 500 µs, with 400 µs used in five studies. One study did not report PW [22]. Stimulation intensity varied from 20 to 41 mA and was adjusted based on visible muscle contraction and patient tolerance. Most studies employed symmetric biphasic waveforms. Detailed descriptions of the NMES protocols and devices are provided in Table 2.

**Table 2:** NMES protocols from the included studies.

Author (Year), country	Frequency (Hz)	Pulse width (µs)	Up‐plateau‐down (s)	On‐off (s)	Amplitude	Sessions	Muscles	Devices
Abdellaoui et al. (2011), France	35	400	—	—	Based on patient tolerance	5 × 60 min/week for 6 weeks	Quadriceps and hamstring	Phenix‐S8; Vivaltis, Lattes, France
Kayambu et al. (2015), Australia	40–50	400	—	12–5	—	1–2 × 30 min/day until ICU discharge	Quadriceps, tibialis anterior and brachioradialis	—
Akar et al. (2017), Turkey	50	—	1.5–6–0.75	—	20–25 mA (based on tolerance)	5×/week	Deltoid and quadriceps bilateral	COMPEX, MI theta PRO, Switzerland
Koutsioumpa et al. (2018), Greece	50	500	—	—	Visual assessment of muscle contraction	60 min/day (Day 4–14)	Quadriceps	EN‐STIM 4, Enraf‐Nonius
Silva et al. (2019), Brazil	100	400	—	5–25	Sufficient to evoke maximal contractions	25 min/day after 24 h of MV until Day 14	Quadriceps, hamstring, tibialis anterior and gastrocnemius	Dualpex 071, Quark Medical, Piracicaba, Brazil
França et al. (2020), Brazil	50	500	2–5–2	1:1	Visual assessment of contraction	One 20 min session	Quadriceps Bilateral	Canal FES Neurodyn‐4, Inbramed, Brazil
Nakanish et al. (2020), Japan	20	400	—	2:3	30 mAp (biceps) 41 mAp (quadriceps)	30 min/day (Day 1–5)	Biceps brachii and quadriceps	Solius, Minato Medical Science, Osaka, Japan
Bao et al. (2022), China	30	300	—	1:4	Based on patient tolerance	2 × 20 min/day for > 7 days until discharge	Gastrocnemius and tibialis anterior	QT‐22T, ITO, Japan
Lo Re et al. (2023), Italia	50	200	1.6–1.6	12‐6	Visual assessment of contraction	2 × 60 min/day for 15 days	Quadriceps, tibialis anterior and gastrocnemius	Physioled
Vieira et al. (2023), Brazil	50	400	—	6–12	Sufficient to evoke maximal contractions	55 min/day for 5 days	Quadriceps	Dualpex 071, Quark Medical, Piracicaba, Brazil

### 3.3. Outcomes

All included studies assessed biological biomarkers; however, only five designated biomarkers as primary outcomes [19, 21, 22, 26, 28]. Among the biological markers, inflammatory mediators were analyzed in five studies [19–22, 25]; neuroprotection markers in one [26]; oxidative stress markers in two [19, 28]; and markers related to exercise safety, myopathy, and proteolysis in five [23–25, 27, 28].

Nine studies analyzed biomarkers through blood collection, while one [28] used muscle biopsy. All studies performed pre‐ and post‐intervention assessments. NMES was found to reduce oxidative stress by interfering with monocyte nitric oxide production [19] and by reducing total protein carbonylation and MHC expression [28]. When combined with active mobilization, NMES reduced IL‐6 and IL‐8 levels [22], increased IL‐10^25^, and as standalone therapy, reduced both IL‐8 and C‐reactive protein (CRP) [22]. It also increased IL‐6 when combined with standard rehabilitation [26]. In association with passive cycling, NMES reduced TNF‐α levels [19]. However, two studies with NMES applied in isolation found no significant changes in several cytokines, including TNF‐α, IFN‐γ, IL‐6, IL‐4, IL‐2, TGF‐β, IGF‐1, IL‐1β, MMP‐2, MMP‐9, Klotho, BNDF, FGF23, and IL‐6 [20, 21]. To assess the safety of NMES, studies evaluated lactate levels [23, 25], prothrombin [23], CRP [22, 23, 27], creatine kinase (CK), glucose [27], and amino acid levels [24]. Reductions in amino acid levels were interpreted as indicative of attenuated proteolysis after NMES application.

### 3.4. Effects From RCTs

Due to heterogeneity in study designs, each biomarker was analyzed separately. Analyses considered the timing of outcome measurement, including both acute effects (pre‐ and post‐single session) and effects after a defined intervention period. Day 7 was the most common evaluation point; thus, meta‐analyses prioritized this time frame. When unavailable, post‐intervention values were used [22, 26, 27]. The study by Kayambu et al. [25] was included in both the acute and post‐intervention analyses, as it provided data for both time points.

**Figure 2:**
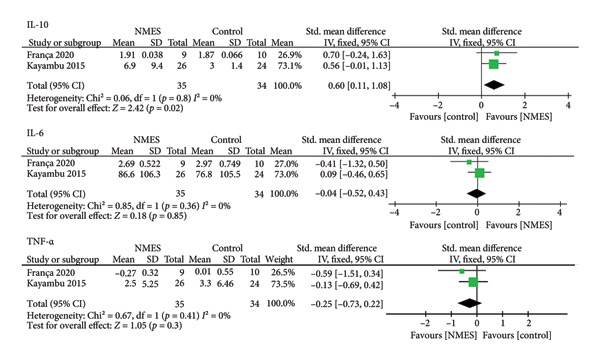
Forest plots comparing the effects of NMES and control interventions on inflammatory biomarkers.

**Figure 3:**
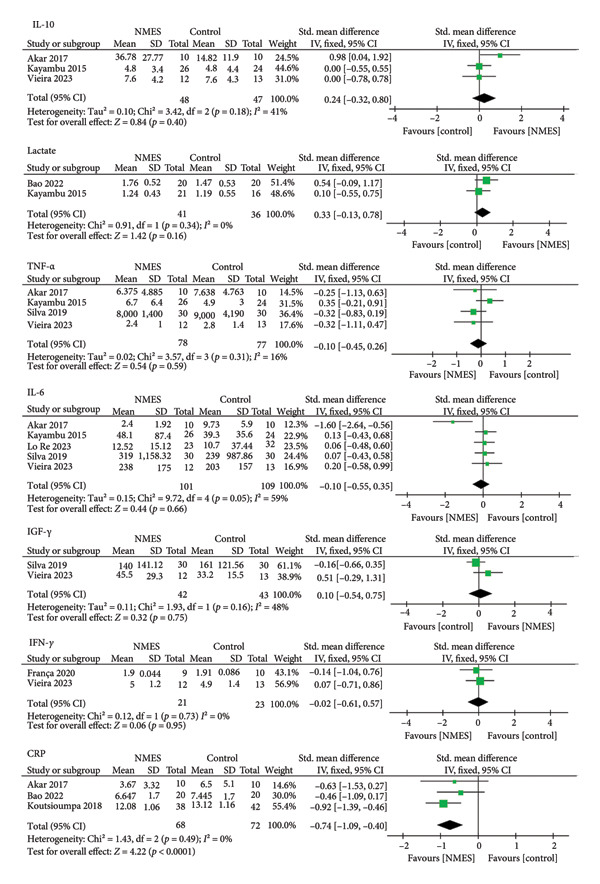
Forest plots of the late‐phase effects of NMES versus control on biological markers.

A meta‐analysis was conducted to synthesize the effects of NMES on biomarkers in critically ill patients. The results demonstrated a significant reduction in CRP levels (SMD: −0.74; 95% CI: −1.09 to −0.40; *p* < 0.0001) and an increase in IL‐10 levels (SMD: 0.60; 95% CI: 0.11 to 1.08; *p* = 0.02) in the acute effect analysis. No significant differences were observed for IL‐6, TNF‐α, IGF‐1, IFN‐γ, or lactate. A random‐effects model was used due to the small number of included studies and high heterogeneity in rehabilitation protocols (Figures 2 and 3). IL‐8 was excluded from meta‐analysis due to limited data.

#### 3.4.1. IL‐10

Four studies reported IL‐10 levels. Two assessed the acute effect (Figure 2), while three evaluated changes from baseline to day 7 (Figure 3). A significant increase in IL‐10 was observed in the acute effect analysis (SMD: 0.60; 95% CI: 0.11 to 1.08; *p* = 0.02).

#### 3.4.2. CRP

Three studies provided data on CRP. A significant reduction in CRP levels over 7 days was observed in the NMES group (SMD: −0.74; 95% CI: −1.09 to −0.40; *p* < 0.0001) (Figure 3).

#### 3.4.3. TNF‐α

Five studies reported TNF‐α levels. Two analyzed the acute effect (Figure 2), and four assessed changes from day 1 to 7 (Figure 3). No significant differences were found between groups.

#### 3.4.4. IGF‐1

Two studies assessed IGF‐1 levels over a 7‐day period. No significant differences were observed (Figure 3).

#### 3.4.5. IFN‐γ

Two studies evaluated IFN‐γ levels. No significant differences were found in the meta‐analysis over 7 days (Figure 3).

#### 3.4.6. IL‐6

Six studies reported IL‐6 levels. Two assessed the acute effect (Figure 2), and five analyzed the intervention effect from day 1 to 7 (Figure 3). No significant changes were detected between groups.

#### 3.4.7. Lactate

Two studies examined lactate levels (Figure 3). No significant differences were observed between intervention and control groups.

### 3.5. Risk of Bias

**Figure 4:**
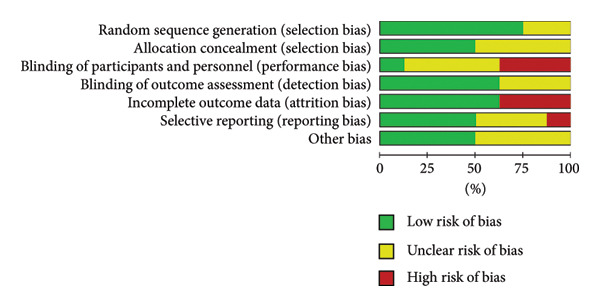
Proportion of studies rated as low, unclear, or high risk of bias across each domain.

**Figure 5:**
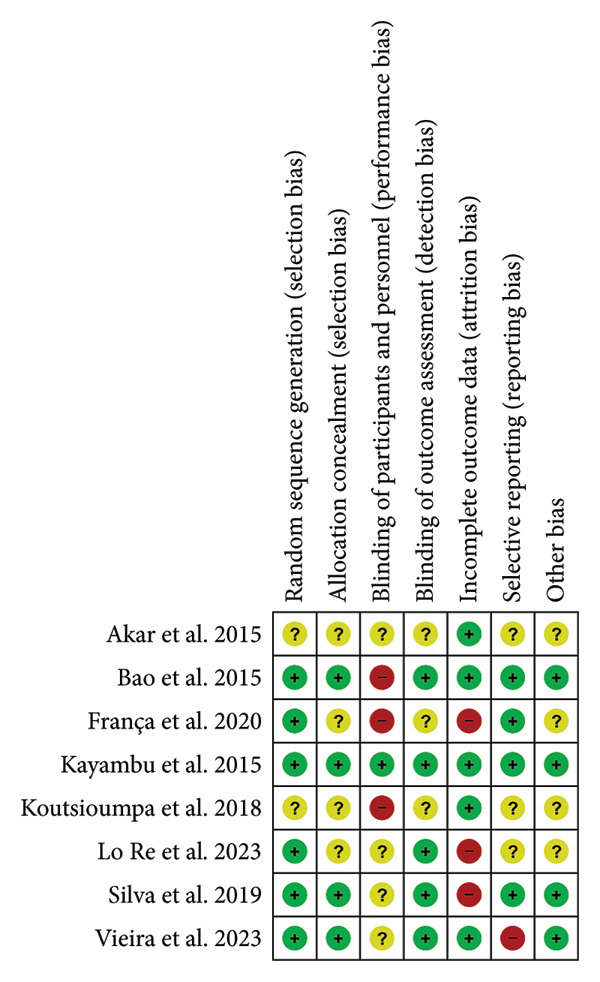
Risk of bias assessment for each domain across individual studies.

All included studies were independently assessed for risk of bias using the Cochrane Risk of Bias Tool for RCTs. Figures 4 and 5 summarize the risk of bias by domain. Overall, most domains presented a low risk of bias. However, high risk was noted for performance bias (three studies), attrition bias (three studies), and reporting bias (one study).

### 3.6. GRADE Assessment

The GRADE assessment identified low‐quality evidence for the acute increase in IL‐10 levels associated with NMES (SMD: 0.60; 95% CI: 0.11 to 1.08; *p* = 0.02). For the reduction in CRP levels observed post‐intervention (SMD: −0.74; 95% CI: −1.09 to −0.40; *p* < 0.0001), the certainty of evidence was rated as very low. All other meta‐analyses also yielded very low‐quality evidence according to GRADE criteria. These ratings were primarily attributed to serious imprecision (due to small sample sizes), indirectness (variability in timing of outcome assessment), and high risk of bias in several included studies (Supporting Information (available here)).

## 4. Discussion

This systematic review analyzed the effects of NMES on biological markers in critically ill patients to elucidate the mechanisms by which the risk of muscle dysfunction risks may be mitigated. Ten RCTs were identified, involving patients with distinct clinical conditions such as COPD, respiratory failure, TBI, post‐cardiac surgery, sepsis, and myopathy, corroborating the findings of Balke et al. [1]. All patients required mechanical ventilation, indicating severe respiratory failure, need for advanced life support, limited mobility, and prolonged bed rest.

In conditions such as sepsis, characterized by complex pathophysiological interactions, it is unlikely that a single biomarker can adequately predict prognosis. Instead, a panel of markers reflecting multiple biological pathways may offer more accurate prognostic value [29, 30]. Accordingly, the meta‐analysis showed that NMES, applied as an intervention to preserve muscle function in ICU patients, exerted immunomodulatory effects by reducing CRP levels over a 7‐day period and increasing IL‐10 levels immediately after the intervention when compared to control groups.

Among inflammatory markers, CRP stands out as a widely used indicator of disease severity and mortality [31, 32]. The meta‐analysis demonstrated that NMES significantly reduced serum CRP levels in critically ill individuals. Although statistical heterogeneity was low (*I* ^2^ = 0%) across the three studies assessing CRP, variations in intervention protocols were evident. Two studies applied NMES five times per week across multiple muscle groups [22, 23], while Koutsioumpa et al. [27] employed stimulation of a single muscle group during 60‐min sessions.

Persistent elevation of CRP may indicate failure to control infection and systemic inflammation, and it has been proposed as a parameter for assessing patient safety [23, 33]. Moreover, elevated circulating levels of inflammatory cytokines, including IL‐6, CRP, and TNF‐α, have been associated with reduced muscle mass and strength in critically ill adults [34]. Thus, a decline in CRP following NMES suggests enhanced control of systemic inflammation, potentially preventing prolonged immunosuppression and supporting recovery. However, it is important to note that CRP is a nonspecific marker influenced by multiple physiological and pathological processes [35].

In addition to CRP reduction, the meta‐analysis demonstrated an acute increase in IL‐10 levels following NMES in ICU patients. Witteveen et al. [36] highlighted that systemic inflammation intensifies in the early phase of ICU admission, with IL‐10 emerging as a pivotal cytokine in the pathophysiology of ICU‐AW. Produced by lymphocytes and monocytes, IL‐10 is a key anti‐inflammatory mediator that attenuates TNF‐α‐driven damage during septic states. Consequently, IL‐10 has been proposed as a potential therapeutic target [37, 38].

An early elevation in IL‐10 may attenuate an excessive proinflammatory response that, while necessary for pathogen clearance, can become dysregulated and result in systemic complications or early mortality. IL‐10 helps prevent hyperactivation of the innate immune system by inhibiting the effects of cytokines such as TNF‐α, IL‐1β, and IL‐6 [39], thereby reducing the risk of multiple organ failure [40], and mitigating complications related to sepsis [41].

Several studies have documented increased IL‐10 levels following acute exercise. For instance, Carvalho et al. [42] reported a rise in serum IL‐10 in critically ill patients undergoing passive cycle ergometry. Similarly, Winkelman et al. [43] demonstrated that longer durations of acute exercise (15–60 min) yielded more favorable immunological responses compared to shorter sessions (5–25 min). These findings are relevant for interpreting the results of França et al. [19], which did not observe a significant IL‐10 increase—likely due to the shorter NMES duration (20 min) applied in their protocol. This suggests that longer NMES sessions (30–60 min) may be required to achieve measurable anti‐inflammatory effects.

With respect to late‐phase effects, the study by Kayambu et al. [25]—also included in our meta‐analysis—showed a significant IL‐10 increase in septic patients subjected to early physical rehabilitation, including 30‐min NMES sessions throughout ICU stay. These findings support the use of NMES as a strategy to modulate inflammation in critical illness. Given the association between IL‐10 and ICU‐AW, our meta‐analysis indicates that NMES acutely elevates IL‐10 levels—a cytokine known for its potent anti‐inflammatory role. Nevertheless, a meta‐analysis of muscle strength outcomes was not feasible due to limited data availability. Future studies should explore the relationship between IL‐10 elevation and improvements in functional parameters such as muscle strength post‐NMES.

Another pertinent issue is the absence of established thresholds regarding the minimal contraction level or session frequency required to trigger myokine release. A dose‐response relationship between NMES intensity and biological effects is plausible, yet still unclear. Additional studies are necessary to elucidate how NMES parameters, such as intensity, frequency, duration, and targeted muscles, interact with the release of myokines [44]. The included studies stimulated different muscle groups and adopted heterogeneous NMES parameters (e.g., frequency, PW, intensity, duration), and there remains no consensus on the optimal protocol to prevent ICU‐related muscle atrophy [45].

According to the systematic review by Balke et al. [1], NMES sessions should ideally last 50–60 min per day, with a 45% duty cycle, corresponding to approximately 25 min of effective contraction time. This review recommends wide pulse and high‐frequency stimulation for the quadriceps muscle, specifically using a frequency of 50 Hz and a PW of 375 µs. Stimulation intensity should be sufficient to elicit visible contractions, typically starting at ≥ 50 mA and not exceeding 100 mA. In our review, most trials adopted similar parameters, particularly a 50‐Hz frequency and a 400‐µs PW. Nonetheless, the optimal configuration of stimulation parameters remains undefined, underscoring the need for further studies to determine the most effective NMES settings in critically ill populations.

Inflammatory processes in critically ill patients may present acutely or chronically, both phases being marked by elevated cytokines such as IL‐1β, TNF‐α, and IL‐6. IL‐6, secreted by macrophages, T lymphocytes, and multiple tissue types, exhibits dual biological roles: it can either exacerbate or mitigate inflammation. Importantly, effective muscle contraction is known to stimulate IL‐6 production, which contributes to the regulation of inflammatory processes in adipose tissue and promotes muscle repair. Moreover, IL‐6 facilitates the expression of IL‐10 receptors, thereby exerting anti‐inflammatory actions [46–48].

Although muscle‐derived IL‐6 is classified as a myokine with systemic endocrine effects [49], its plasma concentrations are often elevated in critically ill individuals [50] particularly during the acute phase of sepsis [51]. In this context, IL‐6 assumes a predominantly proinflammatory profile, being released in response to infection or tissue injury [52]. Consequently, in the presence of multiple concurrent inflammatory stimuli, its clinical specificity as a biomarker becomes limited [53]. In our analysis of seven studies that reported IL‐6 levels, no significant changes were observed following NMES, whether assessed acutely or over time. These findings suggest that NMES may not exert a measurable effect on IL‐6 dynamics in critically ill patients, at least within the evaluation windows employed in the included studies.

TNF‐α is another cytokine of major relevance in innate immunity, playing a central role in orchestrating the inflammatory response. It promotes the recruitment of neutrophils and lymphocytes to the site of inflammation, serving as a key mediator in host defense [54]. Sustained elevations of TNF‐α may reflect a hyperactive immune response to a pathogenic insult. However, the studies included in this review found no significant changes in TNF‐α levels following NMES application, in either acute or subacute assessments. Further investigations are warranted to clarify the circumstances under which NMES might influence TNF‐α regulation.

An important methodological aspect to consider is the short half‐life of many inflammatory cytokines, which challenges their accurate measurement in serum or plasma. TNF‐α, for instance, has a half‐life of approximately 20 min in serum [55]. As such, delays between NMES applications, biological sampling, and analysis may result in underestimated cytokine concentrations, thereby limiting the interpretability of results.

It is also plausible that patient‐related factors, such as the severity of the inflammatory or infectious process, age, sex, and sampling timing, contributed to the lack of significant changes in IGF‐1 and IFN‐γ observed in the reviewed studies. Regarding NMES safety, few studies assessed biomarkers specifically related to adverse effects. In addition to CRP, Bao et al. [23] evaluated prothrombin time and lactate as safety markers during NMES. Homma et al. [33] proposed that in elderly patients, especially those undergoing hemodialysis, CK and IL‐6 could serve as markers for monitoring NMES‐related muscle damage and inflammation.

Among laboratory tests for detecting muscle injury, elevated CK is considered a sensitive marker. In the absence of myocardial or cerebral injury, CK values exceeding 5000 U/L are suggestive of severe myocellular damage [56]. In a systematic review on the safety of NMES applications, Kemmler et al. [57] reported no serious adverse events. However, some studies described CK elevations compatible with moderate to severe rhabdomyolysis, particularly when excessive intensities were used during initial sessions. These observations underscore the importance of gradual intensity progression during NMES to mitigate the risk of muscle injury.

Histopathological evidence has also shown that NMES can induce structural alterations in muscle fibers and connective tissue, leading to increased serum CK activity [58]. In rhabdomyolysis, muscle cell damage triggers membrane disruption and the release of intracellular components, notably myoglobin and CK, into systemic circulation [59]. Despite their clinical relevance, only one study included in this review assessed CK levels, revealing an important gap in the current literature. Future studies on NMES should incorporate both CK and myoglobin to enhance the safety profile assessment of this modality.

The findings of this review indicate that NMES in critically ill patients produces systemic effects that include immunological modulation. In the early phase, these effects are reflected by an immediate rise in IL‐10 levels, while in later stages, they manifest as reductions in persistent inflammation, evidenced by decreased CRP levels. These outcomes support the safety of NMES even during active disease phases within ICU settings, environments characterized by high susceptibility to inflammatory complications, particularly among immobile patients unable to perform active exercise.

Furthermore, the present results reveal important gaps in the literature that must be addressed. Specifically, there is limited knowledge regarding which other biological markers are influenced by NMES and how these responses evolve temporally. Clarifying these interactions will be essential to understanding their potential associations with functional and clinical outcomes, including muscle strength, mechanical ventilation duration, weaning success, ICU length of stay, prevalence of ICU‐AW, readmission rates, and overall patient prognosis. Addressing these evidence gaps will contribute to the development of refined and evidence‐based intervention protocols, as well as comprehensive biomarker‐based monitoring strategies.

Despite the potential benefits of NMES in critically ill populations, this review identified several methodological limitations across studies, including high risk of bias, small sample sizes, lack of protocol standardization (including stimulation parameters and devices), and inconsistent biomarker selection. Such heterogeneity impaired the ability to perform broader meta‐analyses and hindered the identification of optimal stimulation protocols. Moreover, the scarcity of studies specifically addressing biomarker responses to NMES in ICU settings highlights the need for more robust, well‐designed clinical trials in this field.

## 5. Conclusion

NMES, when employed to preserve or restore muscle strength in critically ill patients undergoing invasive mechanical ventilation and prolonged immobility, modulates biological markers associated with systemic inflammation. The evidence synthesized in this review indicates that NMES induces an immediate increase in IL‐10 levels and, after seven days of intervention, promotes a reduction in CRP levels compared to control conditions.

These findings support the safety of NMES, even during the acute phase of critical illness, and underscore the importance of conducting further RCTs with standardized stimulation protocols and careful consideration of the pharmacokinetics and half‐lives of inflammatory biomarkers. Such investigations are essential to deepen the understanding of NMES‐induced immunomodulatory mechanisms and to guide its implementation in clinical practice.

## Disclosure

All authors read and approved the final manuscript. This manuscript is based on the first author’s master’s thesis titled “Impact of neuromuscular electrical stimulation on biological markers in critically ill patients: a systematic review and meta‐analysis” [60] presented to the Federal University of Sergipe (UFS) in 2024.

## Conflicts of Interest

The authors declare no conflicts of interest.

## Author Contributions

All authors participated in the research and preparation of the manuscript. Methodology: Amanda de Oliveira Santos, Maíra Ávila Fontes Trindade, and Érika Ramos Silva; original draft writing: Amanda de Oliveira Santos, Érika Ramos Silva, and Matheus Cardoso Santos; formal analysis: Amanda de Oliveira Santos and Matheus Cardoso Santos; data extraction and search: Amanda de Oliveira Santos, Danielle Alves de Andrade Rebouças, Fernanda Oliveira de Carvalho, and Érika Ramos Silva; writing–review and editing: Maíra Ávila Fontes Trindade, Carlos José Oliveira de Matos, Fernanda Oliveira de Carvalho, and Paulo Ricardo Martins‐Filho; risk‐of‐bias and quality assessment: Amanda de Oliveira Santos and Danielle Alves de Andrade Rebouças; project administration: ERS.

## Funding

This work was partially supported by IReL ‐ ACS Exclusive Contact based on an agreement between IReL—ACS Exclusive Contact and Wiley.

## Supporting Information

Supporting File 1. Systematic Review Protocol: Detailed protocol outlining the methodology for the systematic review and meta‐analysis on the effects of neuromuscular electrical stimulation in critically ill patients. Includes eligibility criteria, search strategy, data extraction, risk of bias assessment, meta‐analysis plan, GRADE evidence summary, and a table of excluded full‐text articles with reasons.

## Supporting information


**Supporting Information** Additional supporting information can be found online in the Supporting Information section.

## Data Availability

The data that support the findings of this study are available from the corresponding author upon reasonable request.

## References

[1] Balke M. , Teschler M. , Schäfer H. , Pape P. , Mooren F. C. , and Schmitz B. , Therapeutic Potential of Electromyostimulation (EMS) in Critically Ill Patients—A Systematic Review, Frontiers in Physiology. (2022) 13, 10.3389/fphys.2022.865437. PMC9124773 35615672

[2] Fazzini B. , Märkl T. , Costas C. et al., The Rate and Assessment of Muscle Wasting During Critical Illness: A Systematic Review and Meta-Analysis, Critical Care. (2023) 27, no. 1, 10.1186/s13054-022-04253-0. PMC9808763 36597123

[3] Xu Q. , Tan J. , Wang Y. , and Tang M. , Theory-Based and Evidence-Based Nursing Interventions for the Prevention of ICU-Acquired Weakness in the Intensive Care Unit: A Systematic Review, PLoS One. (2024) 19, no. 9, 10.1371/journal.pone.0308291. PMC11398680 39269947

[4] Song J. , Deng T. , Yu Q. et al., Biomarkers for Intensive Care Unit-Acquired Weakness: A Systematic Review for Prediction, Diagnosis and Prognosis, Annals of Intensive Care. (2025) 15, no. 1, 10.1186/s13613-025-01500-9. PMC12222590 40601169

[5] Chen X. X. , Xiong J. , Chen J. X. et al., Trajectory and Determinants of Intensive Care Unit–Acquired Weakness in Critical Illness: A Multicentre, Prospective, Longitudinal Study, Nursing in Critical Care. (2024) 30, no. 4, 10.1111/nicc.13209. 39632248

[6] Ribeiro L. C. , Amaral T. C. N. , Vilaça A. F. et al., Acute Effect of Neuromuscular Electrical Stimulation on Oxidative Stress and Hematological Parameters in Critical Patients, Journal of Immunobiology. (2017) 02, no. 03, 10.4172/2476-1966.1000131.

[7] Zayed Y. , Kheiri B. , Barbarawi M. et al., Effects of Neuromuscular Electrical Stimulation in Critically Ill Patients: A Systematic Review and Meta-Analysis of Randomised Controlled Trials, Australian Critical Care. (2020) 33, no. 2, 203–210, 10.1016/j.aucc.2019.04.003, 2-s2.0-85066326743. 31160215

[8] Othman S. Y. , Elbiaa M. A. , Mansour E. R. , El‐Menshawy A. M. , and Elsayed S. M. , Effect of Neuromuscular Electrical Stimulation and Early Physical Activity on ICU‐Acquired Weakness in Mechanically Ventilated Patients: A Randomized Controlled Trial, Nursing in Critical Care. (2024) 29, no. 3, 584–596, 10.1111/nicc.13010. 37984373

[9] Burke D. , Gorman E. , Stokes D. , and Lennon O. , An Evaluation of Neuromuscular Electrical Stimulation in Critical Care Using the ICF Framework: A Systematic Review and Meta‐Analysis, The Clinical Respiratory Journal. (2016) 10, no. 4, 407–420, 10.1111/crj.12234, 2-s2.0-84978648949. 25353646

[10] Alqurashi H. B. , Robinson K. , O’Connor D. et al., The Effects of Neuromuscular Electrical Stimulation on Hospitalised Adults: Systematic Review and Meta-Analysis of Randomised Controlled Trials, Age and Ageing. (2023) 52, no. 12, 10.1093/ageing/afad236. PMC10756181 38156975

[11] Wahl P. , Hein M. , Achtzehn S. , Bloch W. , and Mester J. , Acute Effects of Superimposed Electromyostimulation During Cycling on Myokines and Markers of Muscle Damage, Journal of Musculoskeletal and Neuronal Interactions. (2015) 15, no. 1, 53–59. 25730652 PMC5123608

[12] Grunow J. J. , Goll M. , Carbon N. M. , Liebl M. E. , Weber-Carstens S. , and Wollersheim T. , Differential Contractile Response of Critically Ill Patients to Neuromuscular Electrical Stimulation, Critical Care. (2019) 23, no. 1, 10.1186/s13054-019-2540-4, 2-s2.0-85072044097. PMC6737711 31506074

[13] Lago A. F. , Basile-Filho A. , de Oliveira A. S. , de Souza H. C. D. , Dos Santos D. O. , and Gastaldi A. C. , Effects of Physical Therapy With Neuromuscular Electrical Stimulation in Acute and Late Septic Shock Patients: A Randomised Crossover Clinical Trial, PLoS One. (2022) 17, no. 2, 10.1371/journal.pone.0264068. PMC8853464 35176099

[14] Moher D. , Liberati A. , Tetzlaff J. , and Altman D. G. , Preferred Reporting Items for Systematic Reviews and Meta-Analyses: The PRISMA Statement, BMJ. (2009) 339, no. 1, 10.1136/bmj.b2535, 2-s2.0-69449100622. PMC3090117 21603045

[15] Rohatgi A. , Webplotdigitizer, 2017.

[16] Higgins J. P. , Altman D. G. , Gøtzsche P. C. et al., The Cochrane Collaboration’s Tool for Assessing Risk of Bias in Randomised Trials, BMJ. (2011) 343, no. 2, 10.1136/bmj.d5928, 2-s2.0-84859001212. PMC3196245 22008217

[17] Higgins J. P. , Thompson S. G. , Deeks J. J. , and Altman D. G. , Measuring Inconsistency in Meta-Analyses, BMJ. (2003) 327, no. 7414, 557–560, 10.1136/bmj.327.7414.557. 12958120 PMC192859

[18] Guyatt G. , Oxman A. D. , Akl E. A. et al., GRADE Guidelines: 1. Introduction—GRADE Evidence Profiles and Summary of Findings Tables, Journal of Clinical Epidemiology. (2011) 64, no. 4, 383–394, 10.1016/j.jclinepi.2010.04.026, 2-s2.0-79951952372. 21195583

[19] França E. E. T. , Gomes J. P. V. , De Lira J. M. B. et al., Acute Effect of Passive Cycle-Ergometry and Functional Electrical Stimulation on Nitrosative Stress and Inflammatory Cytokines in Mechanically Ventilated Critically Ill Patients: A Randomized Controlled Trial, Brazilian Journal of Medical and Biological Research. (2020) 53, no. 4, 10.1590/1414-431X20208770. PMC7162584 32294698

[20] Silva P. E. , de Cássia Marqueti R. , Livino-de-Carvalho K. et al., Neuromuscular Electrical Stimulation in Critically Ill Traumatic Brain Injury Patients Attenuates Muscle Atrophy, Neurophysiological Disorders, and Weakness: A Randomized Controlled Trial, Journal of Intensive Care. (2019) 7, no. 1, 10.1186/s40560-019-0417-x. PMC6909464 31890221

[21] Vieira L. , Silva P. E. , de Melo P. F. et al., Early Neuromuscular Electrical Stimulation Preserves Muscle Size and Quality and Maintains Systemic Levels of Signaling Mediators of Muscle Growth and Inflammation in Patients With Traumatic Brain Injury: A Randomized Clinical Trial, Critical care research and practice. (2023) 2023, no. 1, 10.1155/2023/9335379. PMC10397495 37547450

[22] Akar O. , Günay E. , Sarinc Ulasli S. et al., Efficacy of Neuromuscular Electrical Stimulation in Patients With COPD Followed in Intensive Care Unit, The Clinical Respiratory Journal. (2017) 11, no. 6, 743–750, 10.1111/crj.12411, 2-s2.0-84950335755. 26597394

[23] Bao W. , Yang J. , Li M. et al., Prevention of Muscle Atrophy in ICU Patients Without Nerve Injury by Neuromuscular Electrical Stimulation: A Randomized Controlled Study, BMC Musculoskeletal Disorders. (2022) 23, no. 1, 10.1186/s12891-022-05739-2. PMC9380284 35974369

[24] Nakanishi N. , Oto J. , Tsutsumi R. et al., Effect of Electrical Muscle Stimulation on Upper and Lower Limb Muscles in Critically Ill Patients: A Two-Center Randomized Controlled Trial, Critical Care Medicine. (2020) 48, no. 11, e997–e1003, 10.1097/CCM.0000000000004522. 32897665

[25] Kayambu G. , Boots R. , and Paratz J. , Early Physical Rehabilitation in Intensive Care Patients With Sepsis Syndromes: A Pilot Randomised Controlled Trial, Intensive Care Medicine. (2015) 41, no. 5, 865–874, 10.1007/s00134-015-3763-8, 2-s2.0-84937763252. 25851383

[26] Lo Re V. , Russelli G. , Lo Gerfo E. et al., Cognitive Outcomes in Patients Treated With Neuromuscular Electrical Stimulation After Coronary Artery Bypass Grafting, Frontiers in Neurology. (2023) 14, 10.3389/fneur.2023.1209905. PMC10486105 37693766

[27] Koutsioumpa E. , Makris D. , Theochari A. et al., Effect of Transcutaneous Electrical Neuromuscular Stimulation on Myopathy in Intensive Care Patients, American Journal of Critical Care. (2018) 27, no. 6, 495–503, 10.4037/ajcc2018311, 2-s2.0-85055910981. 30385541

[28] Abdellaoui A. , Préfaut C. , Gouzi F. et al., Skeletal Muscle Effects of Electrostimulation After COPD Exacerbation: A Pilot Study, European Respiratory Journal. (2011) 38, no. 4, 781–788, 10.1183/09031936.00167110, 2-s2.0-80053056079. 21349913

[29] Griffith D. M. , Lewis S. , Rossi A. G. et al., Systemic Inflammation After Critical Illness: Relationship With Physical Recovery and Exploration of Potential Mechanisms, Thorax. (2016) 71, no. 9, 820–829, 10.1136/thoraxjnl-2015-208114, 2-s2.0-84965031273. 27118812

[30] Méndez Hernández R. and Ramasco Rueda F. , Biomarkers as Prognostic Predictors and Therapeutic Guide in Critically Ill Patients: Clinical Evidence, Journal of Personalized Medicine. (2023) 13, no. 2, 10.3390/jpm13020333. PMC9965041 36836567

[31] Lavillegrand J. R. , Garnier M. , Spaeth A. et al., Elevated Plasma IL-6 and CRP Levels are Associated With Adverse Clinical Outcomes and Death in Critically Ill SARS-CoV-2 Patients: Inflammatory Response of SARS-CoV-2 Patients, Annals of Intensive Care. (2021) 11, no. 1, 10.1186/s13613-020-00798-x. PMC7804215 33439360

[32] Zhang Z. and Ni H. , C-Reactive Protein as a Predictor of Mortality in Critically Ill Patients: A Meta-Analysis and Systematic Review, Anaesthesia & Intensive Care. (2011) 39, no. 5, 854–861, 10.1177/0310057X1103900509. 21970129

[33] Homma M. , Miura M. , Hirayama Y. et al., Belt Electrode-Skeletal Muscle Electrical Stimulation in Older Hemodialysis Patients With Reduced Physical Activity: A Randomized Controlled Pilot Study, Journal of Clinical Medicine. (2022) 11, no. 20, 10.3390/jcm11206170. PMC9605129 36294490

[34] Tuttle C. S. , Thang L. A. , and Maier A. B. , Markers of Inflammation and Their Association With Muscle Strength and Mass: A Systematic Review and Meta-Analysis, Ageing Research Reviews. (2020) 64, 10.1016/j.arr.2020.101185. 32992047

[35] Heilmann E. , Gregoriano C. , and Schuetz P. , Biomarkers of Infection: Are They Useful in the ICU? Seminars in Respiratory and Critical, Care Medicine. (2019) 40, no. 04, 465–475, 10.1055/s-0039-1696689, 2-s2.0-85072917216. PMC7117078 31585473

[36] Witteveen E. , Wieske L. , van der Poll T. et al., Increased Early Systemic Inflammation in ICU-Acquired Weakness; A Prospective Observational Cohort Study, Critical Care Medicine. (2017) 45, no. 6, 972–979, 10.1097/CCM.0000000000002408, 2-s2.0-85016122993. 28350642

[37] Ward N. S. , Casserly B. , and Ayala A. , The Compensatory Anti-Inflammatory Response Syndrome (CARS) in Critically Ill Patients, Clinics in Chest Medicine. (2008) 29, no. 4, 617–625, 10.1016/j.ccm.2008.06.010, 2-s2.0-54049130105. 18954697 PMC2786900

[38] Kim P. K. and Deutschman C. S. , Inflammatory Responses and Mediators, Surgical Clinics of North America. (2000) 80, no. 3, 885–894, 10.1016/S0039-6109(05)70102-X, 2-s2.0-0034123347. 10897267

[39] Inoue G. , Effect of Interleukin-10 (IL-10) on Experimental LPS-Induced Acute Lung Injury, Journal of Infection and Chemotherapy. (2000) 6, no. 1, 51–60, 10.1007/s101560050050, 2-s2.0-0034095250. 11810532

[40] Sapan H. B. , Paturusi I. , Jusuf I. et al., Pattern of Cytokine (IL-6 and IL-10) Level as Inflammation and Anti-Inflammation Mediator of Multiple Organ Dysfunction Syndrome (MODS) in Polytrauma, International Journal of Burns and Trauma. (2016) 6, no. 2, 37–43. 27335696 PMC4913232

[41] Li J. , Xiao C. , and Zheng H. , Prognostic Value of Inflammatory Cytokine Detection for Sepsis Patients in ICU: A Meta-Analysis, American Journal of Tourism Research. (2024) 16, no. 6, 2612–2621, 10.62347/nylm7723. PMC11236661 39006300

[42] Carvalho M. T. X. , Real A. A. , Cabeleira M. E. et al., Acute Effect of Passive Cycling Exercise on Serum Levels of Interleukin-8 and Interleukin-10 in Mechanically Ventilated Critically Ill Patients, International Journal of Therapy and Rehabilitation. (2020) 27, no. 9, 1–7, 10.12968/ijtr.2018.0141.

[43] Winkelman C. , Johnson K. D. , Hejal R. et al., Examining the Positive Effects of Exercise in Intubated Adults in ICU: A Prospective Repeated Measures Clinical Study, Intensive and Critical Care Nursing. (2012) 28, no. 6, 307–318, 10.1016/j.iccn.2012.02.007, 2-s2.0-84869088687. 22458998 PMC3783509

[44] Sanchis-Gomar F. , Lopez-Lopez S. , Romero-Morales C. , Maffulli N. , Lippi G. , and Pareja-Galeano H. , Neuromuscular Electrical Stimulation: A New Therapeutic Option for Chronic Diseases Based on Contraction-Induced Myokine Secretion, Frontiers in Physiology. (2019) 10, 10.3389/fphys.2019.01463. PMC6894042 31849710

[45] Xu C. , Yang F. , Wang Q. , and Gao W. , Effect of Neuromuscular Electrical Stimulation in Critically Ill Adults With Mechanical Ventilation: A Systematic Review and Network Meta-Analysis, BMC Pulmonary Medicine. (2024) 24, no. 1, 10.1186/s12890-024-02854-9. PMC10811936 38273243

[46] da Luz Scheffer D. and Latini A. , Exercise-Induced Immune System Response: Anti-Inflammatory Status on Peripheral and Central Organs, Biochimica et Biophysica Acta-Molecular Basis of Disease. (2020) 1866, no. 10, 10.1016/j.bbadis.2020.165823. PMC7188661 32360589

[47] Marini A. C. , Motobu R. D. , Lobo P. C. , Monteiro P. A. , and Pimentel G. D. , No Effect of Intradialytic Neuromuscular Electrical Stimulation on Inflammation and Quality of Life: A Randomized and Parallel Design Clinical Trial, Scientific Reports. (2021) 11, no. 1, 10.1038/s41598-021-01498-7. PMC8590010 34772982

[48] Pedersen B. K. , Åkerström T. C. , Nielsen A. R. , and Fischer C. P. , Role of Myokines in Exercise and Metabolism, Journal of Applied Physiology. (2007) 103, no. 3, 1093–1098, 10.1152/japplphysiol.00080.2007, 2-s2.0-34548449306. 17347387

[49] Omoto M. , Matsuse H. , Hashida R. et al., Cycling Exercise With Electrical Stimulation of Antagonist Muscles Increases Plasma Growth Hormone and IL-6, Tohoku Journal of Experimental Medicine. (2015) 237, no. 3, 209–217, 10.1620/tjem.237.209, 2-s2.0-84946416852. 26522057

[50] Zanders L. , Kny M. , Hahn A. et al., Sepsis Induces Interleukin 6, gp130/JAK2/STAT3, and Muscle Wasting, Journal of Cachexia, Sarcopenia and Muscle. (2022) 13, no. 1, 713–727, 10.1002/jcsm.12867. 34821076 PMC8818599

[51] Song J. , Park D. W. , Moon S. et al., Diagnostic and Prognostic Value of Interleukin-6, Pentraxin 3, and Procalcitonin Levels Among Sepsis and Septic Shock Patients: A Prospective Controlled Study According to the Sepsis-3 Definitions, BMC Infectious Diseases. (2019) 19, no. 1, 10.1186/s12879-019-4618-7. PMC6852730 31718563

[52] Kang S. and Kishimoto T. , Interplay Between Interleukin-6 Signaling and the Vascular Endothelium in Cytokine Storms, Experimental and Molecular Medicine. (2021) 53, no. 7, 1116–1123, 10.1038/s12276-021-00649-0. 34253862 PMC8273570

[53] Mierzchała-Pasierb M. and Lipińska-Gediga M. , Sepsis Diagnosis and Monitoring–Procalcitonin as Standard, But What Next?, Anaesthesiology Intensive Therapy. (2019) 51, no. 4, 299–305, 10.5114/ait.2019.88104. 31550871

[54] Oberholzer A. , Oberholzer C. , and Moldawer L. L. , Cytokine Signaling-Regulation of the Immune Response in Normal and Critically Ill States, Critical Care Medicine. (2000) 28, no. 4, N3–N12, 10.1097/00003246-200004001-00002. 10807312

[55] Lenz A. , Franklin G. A. , and Cheadle W. G. , Systemic Inflammation After Trauma, Injury. (2007) 38, no. 12, 1336–1345, 10.1016/j.injury.2007.10.003, 2-s2.0-36549019937. 18048040

[56] Huerta-Alardín A. L. , Varon J. , and Marik P. E. , Bench-To-Bedside Review: Rhabdomyolysis–An Overview for Clinicians, Critical Care. (2004) 9, no. 2, 10.1186/cc2978, 2-s2.0-15844385432. PMC1175909 15774072

[57] Kemmler W. , Weissenfels A. , Willert S. et al., Efficacy and Safety of Low Frequency Whole-Body Electromyostimulation (WB-EMS) to Improve Health-Related Outcomes in Non-athletic Adults. A Systematic Review, Frontiers in Physiology. (2018) 9, 10.3389/fphys.2018.00573, 2-s2.0-85047380249. PMC5974506 29875684

[58] Kästner A. , Braun M. , and Meyer T. , Two Cases of Rhabdomyolysis After Training With Electromyostimulation by 2 Young Male Professional Soccer Players, Clinical Journal of Sport Medicine. (2015) 25, no. 6, e71–e73, 10.1097/JSM.0000000000000153. 25353720

[59] Estes M. E. Z. , Rhabdomyolysis After Exercise With an Electrical Muscle Stimulator, The Nurse Practitioner. (2018) 43, no. 9, 8–12, 10.1097/01.NPR.0000544286.79459.19, 2-s2.0-85052243173. 30134431

[60] Santos A. O. , Impact of Neuromuscular Electrical Stimulation on Biological Markers in Critically Ill Patients: A Systematic Review and Meta-Analysis, 2024, Lagarto: Universidade Federal de Sergipe, https://ri.ufs.br/handle/riufs/19841, Master’s Thesis. 10.1155/ccrp/5382735 PMC12820417 41573125

