# Supplementary material for: Impact of Neuromuscular Electrical Stimulation on Biological Markers in Critically Ill Patients: A Systematic Review and Meta‐Analysis
Source: Crit Care Res Pract. 2026 Jan 20;2026:5382735. doi: 10.1155/ccrp/5382735 (PMC12820417; doi:10.1155/ccrp/5382735)
Supplement: Supplementary file 1 — Supporting Information Additional supporting information can be found online in the Supporting Information section. [file CCRP-2026-5382735-s001.docx]

**Supplementary file**

**Systematic Review Protocol**

| **Title of the review** | Impact of Neuromuscular Electrical Stimulation on Biological Markers in Critically Ill Patients: A Systematic Review and Meta-Analysis |
| --- | --- |
| **Authors** | Amanda de Oliveira Santos, Matheus Cardoso Santos, Maíra Ávila Fontes Trindade, Danielle Alves de Andrade Rebouças, Carlos José Oliveira de Matos, Fernanda Oliveira de Carvalho, Paulo Ricardo Martins-Filho, Érika Ramos Silva |
| **Supervisor/Project PI** | Érika Ramos Silva |
| **Institution** | Federal University of Sergipe |

| **1. Background** | |
| --- | --- |
| Patients admitted to intensive care units (ICU) require intensive care, face mobility restrictions, and often necessitate advanced life support interventions. Prolonged bed rest in these patients can lead to Intensive Care Unit Acquired Weakness (ICU-AW), a condition that typically manifests after 24 hours and progresses gradual. Wahl et al. (2015) reported that NMES-induced muscle contraction stimulates muscles to secrete cytokines, contributing to muscle mass gain and skeletal muscle tension. This therapy has demonstrated benefits in ICU patients, in hospital settings, post-cardiac surgery, and in progressively debilitating conditions such as Chronic Obstructive Pulmonary Disease (COPD) and Heart Failure (HF). Several studies have investigated the impact of NMES on biological markers of ICU patients; however, further clarification is needed on the effects of this treatment modality. To date, no systematic review with meta-analysis has been conducted to elucidate the effects of NMES on inflammatory markers in critically ill patients.  Aim  This systematic review and meta-analysis aimed to synthesize the available evidence on the impact of NMES on inflammatory markers in critically ill patients. | |
| **2a. Criteria for including studies in the review based on PICOT elements** | |
| P (population) | Critically ill patients |
| I (intervention) | Neuromuscular electrical stimulation (NMES) |
| C (comparison) | Without neuromuscular electrical stimulation undergoing any other physiotherapy motor resource or none |
| O (outcomes) | Biomarkers |
| T (study type) | Randomized controlled trial. |
| **2b. Criteria for excluding studies in the review** | |
| Observational studies, systematic reviews, editorials, book chapters, conference abstracts, pilot studies, case reports. | |
| **3. Search methods** | |
| Electronic databases | EMBASE, PubMed, PEDro, Web of Science, Scopus, and CENTRAL. |
| Other methods used for identifying relevant research | Google Scholar, BDTD and Proquest. |
| Search strategy | ((Critical care) OR (Intensive Care) OR (Surgical Intensive Care) OR (Critical illness) OR (Critical Illnesses) OR (Critically Ill) OR (Intensive Care Unit) OR (ICU) OR (Intensive Care Units) OR (Critical Illness Polyneuropathy) OR (Intensive care unit–acquired weakness) OR (Hospitalization)) AND ((Electrical Stimulation Therapy) OR (Therapeutic Electrical Stimulation) OR (Electrical Stimulation) OR (Neuromuscular stimulation) OR (Neuromuscular electrical stimulation) OR (NMES) OR (Electromyostimulation) OR (Electrostimulation)) AND ((Biomarkers) OR (Biological Marker) OR (Immune Markers) OR (Immunologic Markers) OR (Clinical Marker) OR (Biochemical Marker) OR (Cytokines) OR (Inflammatory markers) OR (Inflammation mediators) OR (Inflammation) OR (Acute phase proteins) OR (C-Reactive Protein) OR (Interleukin-6) OR (Tumor necrosis factor alpha) OR (Creatine Kinase) OR (Lactate dehydrogenase) OR (Blood lactate)) AND ((randomized controlled trial) OR (controlled clinical trial) OR (comparative study) OR (clinical trial) OR (randomized) OR (placebo) OR (drug therapy) OR (randomly) OR (trial) OR (groups)). |
| Language restriction | No |
| Filters | No |
| Search date | February 10, 2025 |
| **4. Methods of review** | |
| Study selection | Two independent investigators (A.O.S and D.A.A.R.) screened the searched studies based on the title and abstract of each paper. Relevant studies were read in full and selected according to the eligibility criteria. Disagreements between the two reviewers were resolved by consensus or by a third reviewer (E.R.S.). |
| Risk of bias assessment | The risk of bias was assessed by two independent researchers (D.A.A.R. and A.O.S) following the Cochrane guidelines for RCTs. Seven domains were assessed: sequence generation and allocation concealment (selection bias), blinding of participants and researchers (performance bias), outcome assessment (detection bias), incomplete outcome data (attrition bias), selective outcome reporting (reporting bias), and other potential sources of bias. The risk of bias was classified as low, uncertain, or high based on predetermined criteria. |
| Data extraction | Two main reviewers. The reviewers must add information to a standardized data extraction worksheet in Excel. The following information must be extracted: study characteristics (e.g., author, country, and year of publication), objectives, intervention and control groups, sample size, participant characteristics (e.g., number of participants, age, and gender), intervention details (e.g., frequency, pulse width, rise time, sustainment, descent, on and off time, intensity, sessions, muscle targeted, devices), analyzed biomarkers, and results. For continuous outcomes: means and standard deviations for each study group. |
| **5. Meta-analysis** | |
| Data synthesis | Continuous variables: standardized mean difference |
| Statistical heterogeneity | I² index |
| Method | Random or fixed-effects model. In the case of heterogeneity, the random effects model will be used |
| Results presentation | Forest plot |
| Software | Review Manager software (RevMan, version 5.4) |
| **6. Strength of evidence** | |
| GRADE system (https://www.gradepro.org/) | |
| *Factors that can reduce the quality of the evidence* | |
| Risk of bias across studies | ↓ 1 or 2 levels |
| Inconsistency of results | ↓ 1 or 2 levels |
| Indirectness of evidence | ↓ 1 or 2 levels |
| Imprecision | ↓ 1 or 2 levels |
| Influence of small trials (< 100 patients) | ↓ 1 or 2 levels |
| *Factors that can increase the quality of the evidence* | |
| Large magnitude of effect | ↑ 1 or 2 levels |
| All plausible confounding would reduce the demonstrated effect or increase the effect if no effect was observed | ↑ 1 level |
| Dose-response gradient | ↑ 1 level |

**eTable 1. Strength of evidence for efficacy and safety of NMES in critically ill patients.**

| **Outcomes** | **Risk of bias** | **Inconsistency** | **Indirectness** | **Imprecision** | **Quality of evidence** |
| --- | --- | --- | --- | --- | --- |
| **IL-6 (follow-up: average 7 days; assessed with: Blood Collection)** | Very Severe | Severe | Severe | Very Severe | ⨁◯◯◯ |
| **CRP (follow-up: average 7 days; assessed with: Blood Collection)** | Very Severe | Not Serious | Not Serious | Severe | ⨁◯◯◯ |
| **Lactate (follow-up: average 7 days; assessed with: Blood Collection)** | Very Severe | Not Serious | Not Serious | Very Severe | ⨁◯◯◯ |
| **TNF-α (follow-up: average 7 days; assessed with: Blood Collection)** | Very Severe | Not Serious | Severe | Very Severe | ⨁◯◯◯ |
| **IFN-y (follow-up: average 7 days; assessed with: Blood Collection)** | Very Severe | Not Serious | Not Serious | Very Severe | ⨁◯◯◯ |
| **IGF-1 (follow-up: average 7 days; assessed with: Blood Collection)** | Very Severe | Not Serious | Not Serious | Very Severe | ⨁◯◯◯ |
| **IL-10 (follow-up: average 7 days; assessed with: Blood Collection)** | Severe | Not Serious | Severe | Severe | ⨁◯◯◯ |
| **TNF-α (evaluated with: Acute Effect)** | Severe | Not Serious | Not Serious | Very Severe | ⨁◯◯◯ |
| **IL-10 (evaluated with: Acute Effect)** | Severe | Not Serious | Not Serious | Severe | ⨁⨁◯◯ |
| **IL-6 (evaluated with: Acute Effect)** | Severe | Not Serious | Not Serious | Very Severe | ⨁◯◯◯ |

IL-6, Interleukin 6. CRP, C-reactive protein. TNF-α, Tumor Necrosis Factor Alpha. IL-10, Interleukin 10.

Certainty: ⨁ very-low; ⨁⨁ low; ⨁⨁⨁ moderate; ⨁⨁⨁⨁ high

**eTable 2. Full-text articles assessed for eligibility.**

| **Articles** | **Exclusion Criteria** |
| --- | --- |
| Abdellaoui et al., 2011  DOI: 10.1183/09031936.00167110 | - |
| Akar et al., 2017  DOI: 10.1111/crj.12411 | - |
| Bao et al., 2022 DOI: 10.1186/s12891-022-05739-2 | - |
| Bruggemann et al., 2017  DOI: 10.1016/j.apmr.2016.12.009 | Control group also used NMES with a different frequency |
| Cerqueira et al., 2022  DOI: 10.1177/02692155211070945 | Outcomes: Lactate levels available were measured at rest and after the 6-min walk test |
| França et al., 2020 DOI: 10.1590/1414-431X20208770 | - |
| Gerovasili et al., 2009  DOI: 10.1378/chest.08-2888 | Study type is not RCT |
| Groehs et al., 2016  DOI: 10.1177/2047487316654025 | Control group also used NMES with a different intensity |
| Homma, et al., 2022  DOI: 10.3390/jcm11206170 | Patients in ambulatory |
| Karavidas et al., 2006  DOI: 10.1097/01.hjr.0000219111.02544.ff | Control group also used NMES with a different intensity |
| Kayambu et al., 2015 DOI: 10.1007/s00134-015-3763-8 | - |
| Kondo et al., 2019  DOI: 10.1002/ehf2.12504 | Control group also used NMES with a different intensity |
| Koutsioumpa et al., 2018 DOI: 10.4037/ajcc2018311 | - |
| Lo Re et al., 2023 DOI: 10.3389/fneur.2023.1209905 | - |
| Marini et al., 2021  DOI: 10.1038/s41598-021-01498-7 | Population: Patients recruited from two HD clinic |
| Nakanishi et al., 2020  DOI: 10.1097/CCM.0000000000004522 | - |
| Sacilloto et al., 2017  DOI: 10.5935/2359-4802.20170064 | Population: Patients in ambulatory of a university hospital |
| Silva et al., 2019  DOI: 10.1186/s40560-019-0417-x | - |
| Silva et al., 2022  DOI: 10.3390/jcdd9120463 | Population: Patients in home setting |
| Vieira et al., 2023  DOI: 10.1155/2023/9335379 | - |
| Vieira et al., 2014  DOI: 10.1016/j.rmed.2013.12.013 | Population: Stable patients with COPD. Private clinic setting. |
